# The Droplet Digital PCR: A New Valid Molecular Approach for the Assessment of *B-RAF* V600E Mutation in Hairy Cell Leukemia

**DOI:** 10.3389/fphar.2016.00363

**Published:** 2016-10-13

**Authors:** Francesca Guerrini, Matteo Paolicchi, Francesco Ghio, Elena Ciabatti, Susanna Grassi, Serena Salehzadeh, Giacomo Ercolano, Maria R. Metelli, Marzia Del Re, Lorenzo Iovino, Iacopo Petrini, Giovanni Carulli, Nadia Cecconi, Martina Rousseau, Giulia Cervetti, Sara Galimberti

**Affiliations:** ^1^Section of Hematology, Department of Clinical and Experimental Medicine, University of PisaPisa, Italy; ^2^UO hematology, Azienda Ospedaliero-Universitaria PisanaPisa, Italy; ^3^GeNoMEC, University of SienaSiena, Italy; ^4^Clinical Pharmacology and Pharmacogenetic Unit, Department of Clinical and Experimental Medicine, University of PisaPisa, Italy; ^5^Section of Pathology, Department of Translational Medicine and New Technologies, University of PisaPisa, Italy

**Keywords:** *B-RAF*, V600E, hairy cell leukemia, digital droplet PCR, immunoglobulins rearrangement, minimal residual disease

## Abstract

Hairy cell leukemia (HCL) is a chronic lymphoproliferative B-cell disorder where the *B-RAF* V600E mutation has been recently detected, as reported for solid neoplasias but not for other B-cell lymphomas. The digital droplet PCR (dd-PCR) is a molecular technique that, without standard references, is able to accurately quantitate DNA mutations. ddPCR could be an useful instrument for the detection of the *B-RAF* V600E mutation in HCL, where the minimal residual disease monitoring is fundamental for planning a patients-targeted treatment in the era of new anti-CD20 and anti-RAF compounds. This retrospective study enrolled 47 patients observed at the Hematology Unit of the University of Pisa, Italy, from January 2005 to January 2014: 27 patients were affected by “classic” HCL, two by the variant HCL (vHCL), and 18 by splenic marginal zone lymphoma (SMZL). The aim of the study was to compare dd-PCR to “classic” quantitative PCR (QT-PCR) in terms of sensitivity and specificity and to demonstrate its possible use in HCL. Results showed that: (1) the sensitivity of dd-PCR is about half a logarithm superior to QT-PCR (5 × 10^-5^ vs. 2.5 × 10^-4^), (2) the specificity of the dd-PCR is comparable to QT-PCR (no patient with marginal splenic lymphoma or HCL variant resulted mutated), (3) its high sensitivity would allow to use dd-PCR in the monitoring of MRD. At the end of treatment, among patients in complete remission, 33% were still MRD-positive by dd-PCR versus 28% by QT-PCR versus 11% by the evaluation of the B-cell clonality, after 12 months, dd-PCR was comparable to QT-PCR and both detected the *B-RAF* mutation in 15% of cases defined as MRD-negative by IgH rearrangement. Moreover, (4) the feasibility and the costs of dd-PCR are comparable to those of QT-PCR. In conclusion, our study supports the introduction of dd-PCR in the scenario of HCL, also during the follow-up.

## Introduction

*B-RAF*, located on the long arm of the chromosome 7 (Kamiyama et al., 1993), is one of the three *RAF* genes *(A-RAF, B-RAF*, and *C-RAF*) whose activation by their respective ligands (i.e., TGF-alpha and EGF) induces the activation of several downstream targets, such as *ERK1/2, AP1*, and *NFAT*, with the consequent pro-proliferative and anti-apoptotic effect (Chong et al., 2003; Flockhart et al., 2009; Grimaldi et al., 2015).

The *B-RAF* gene presents three fundamental domains: (1) CR1, the amino-terminal portion, cysteine-enriched, where *B-RAF* interacts with *RAS*; (2) CR2, with regulatory activity; (3) CR3, the carboxy-terminal domain, with serine-threonine kinase activity. The V600E mutation in exon 15 involves the CR3 domain, leading a constitutive activation of the *B-RAF* signal, with the consequent uncontrolled proliferative signal (Lyons et al., 2001).

This aberrant kinase activity is also sustained by the overexpression of some non-coding miRNA, such as miR-221 and miR-222, whose expression in HCL is strictly related to the *B-RAF* mutation (Ahmadzadeh et al., 2014).

In the recent years, mutations of *B-RAF*, especially the V600E, have been reported in different types of cancer (Davies et al., 2002): in the 50% of melanomas (DePeralta and Boland, 2015; El-Gamal et al., 2016) and papillary thyroid carcinomas (Jiang et al., 2016), in about 10% of colon (Tie and Desai, 2015) and ovarian cancers (Pakneshan et al., 2013), and in rare cases (1-3%) of non-small cell lung cancer (Caparica et al., 2016).

In the 2011, for the first time, it has been reported that the substitution of the adenine with a thymine at the position 1799, causing the change of a valine with a glutamic acid (the V600E mutation), characterizes 100% of the HCL, allowing distinguishing this histotype from other indolent B-cell lymphomas (Tiacci et al., 2011).

Later, other groups confirmed those results: Arcaini et al. (2012) assessed by allele-specific polymerase chain reaction (ASO-PCR) 240 cases affected by mature B-cell lymphoproliferative disorders, including 62 cases of HCL: the *B-RAF*V600E mutation was detected in all HCL cases, thus confirming that this marker is useful for differentiating HCL from other mimicking lymphomas. Indeed, no patients affected by marginal zone lymphoma or Waldenstrom macroglobulinemia carried the *B-RAF* mutation. Also the variant HCL (v-HCL) was unmutated (Arcaini et al., 2012). More recently, mutations of *MAP2K1* have been reported in unmutated HCL, in addition to the “classical” forms carrying the VH4-34 rearrangement (Mason et al., 2016).

Hairy cell leukemia is a rare B-cell, chronic and indolent lymphoproliferative disease characterized by the bone marrow and spleen/lymph nodes infiltration by pathological CD20+, CD103+, CD25+, CD11c, sIg+, CD5-, CD23-, CD10- lymphocytes (Getta et al., 2015).

Purine analogs (cladibrine, pentostatine) have been reported to induce more than 90% of complete hematological responses (CRs), with 10-40% of relapses or progressions at 16 years (Else et al., 2015; Ravandi, 2015). When MRD is detected by immunohistochemistry, the risk of relapse is significantly higher, thus sustaining the predictive role of the MRD assessment also in this disease (Tallman, 2011).

Nevertheless, in the era of molecular biology and new generation sequencing the immunohistochemistry is not the best technique for the MRD evaluation, especially considering that after the introduction of the anti-B cell therapies (anti-CD20, anti-CD22 antibodies) and of the anti-*RAF* compounds (such as vemurafenib), the possibility of detecting the persistent disease even in a phase of clinical remission is fundamental to design more complex therapeutic strategies that would include “pre-emptive” and/or targeted treatments (Kreitman, 2013).

Today, among the available different molecular techniques, a clear statement on which one would be the best one is still lacking. In 2006, 84 samples from 10 HCL patients were tested for MRD using both flow cytometry and ASO-PCR for the heavy chain immunoglobulin (IgH) rearrangement: the ASO-PCR, with a sensitivity of 1 × 10^-6^, confirmed the results obtained with the flow cytometry and allowed detecting MRD in 91% of cases already defined as CR by the morphological analysis and flow cytometry, with a significant correlation with the clinical outcome (Arons et al., 2006).

In 2008, our group treated 27 HCL patients with rituximab after cladribrine; overall response rate after cladribrine was 89%, with 26% of CRs; after rituximab, CR rate increased up to 89%; concomitantly, a progressive increment in the number of molecular remissions was observed, with MRD-negative cases passing from 40% after cladibrine to 70% after rituximab. The 5-year PFS was 83%, and it was not influenced by age, bone marrow infiltration at diagnosis or quality of response to cladribine, but only by the molecular status after rituximab: 30% of cases still MRD-positive after rituximab remained disease-free vs. 100% of those reaching MRD-negativity after rituximab (Cervetti et al., 2008).

In 2013, Burotto et al. showed that bendamustine offered 100% of overall responses with 30% of CRs, and also in this setting MRD predicted the maintenance of response (Burotto et al., 2013).

Because all HCL cases show the B-cell clonality (90% exhibit VH mutation), the IgH evaluation has been long-term considered the technique of choice for the MRD assessment (Miranda et al., 1999; Sausville et al., 2003). Nevertheless, after discovery of the *B-RAF* mutation, the molecular techniques able to identify this mutation appeared as new more promising tools. Among them, the dd-PCR would be suitable for detecting the *B-RAF* V600E mutation at diagnosis and during follow-up of HCL patients.

Firstly used by Vogelstein and Kinzler (1999) for detecting *RAS* mutations, the high sensitivity and specificity of the dd-PCR, represents a very promising molecular approach: in acute myeloid leukemia, *DNMT3A* o *IDH1/2* mutation detected by dd-PCR were comparable to those obtained by the conventional Sanger sequencing (Brambati et al., 2016). In acute lymphoblastic leukemia, mantle cell lymphoma and multiple myeloma, dd-PCR has been employed in the MRD monitoring: results were comparable to those produced by the conventional ASO-PCR in 94% of cases. In addition, in the 27% of cases reported as “positive not quantifiable” by ASO-PCR, dd-PCR allowed their quantification (Drandi et al., 2015). Also in chronic myeloid leukemia dd-PCR showed a higher sensitivity with respect to the conventional PCR (Zagaria et al., 2015); in the ISAV study, dd-PCR, able to detect one *BCR-ABL1*-positive cell out of 10^-7^, was validated for its predictive role in terms of relapses after imatinib discontinuation in patients with undetectable transcript by QT-PCR. dd-PCR showed a significant negative predictive value, superimposable to that of the conventional PCR (Mori et al., 2015). Finally, our group reported that dd-PCR represents a good technique for assessing the *JAK2* V618F mutation in Philadelphia-negative chronic myeloproliferative neoplasias. In this setting, the possibility of detecting and quantitating the mutation at the same time makes dd-PCR a very competitive method, also in terms of costs (Fontanelli et al., 2015).

The purpose of the present study was to evaluate the introduction of dd-PCR as a molecular approach for the *B-RAF* V600E detection and its comparison with the QT-PCR and IgH rearrangement, at diagnosis and during the follow-up of patients affected by HCL. The advantage of adopting a specific and sensitive technique in HCL is obvious: the identification of the *B-RAF* mutation is fundamental for deciding if, how and when starting a further “patient-tailored” strategy, including the possibility of retreating still MRD-positive patients with the anti-CD20 antibodies as “pre-emptive” therapy or using vemurafenib or rituximab-bendamustine in relapsed cases.

## Materials and Methods

### Patients

This retrospective study enrolled 47 patients observed at the Hematology Unit of the University of Pisa, Italy, from January 2005 to January 2014.

Clinical characteristics are detailed in **Tables 1** and **2**: 27 patients were affected by the “classic” HCL, two by the vHCL (**Table 1**), and 18 by SMZL (**Table 2**). These latest patients were used as controls to evaluate the specificity of the methods, because the *B-RAF* V600E mutation has never been detected in this indolent lymphoma subtype.

**Table 1:** Clinical features of the enrolled HCL patients.

HCL patients (29)		n°	%
Age	Median (y) 58 Range (y) 35-78		
Sex	Male Female	21 8	72 28
Splenomegaly	Yes Mild Large	27 22 5	93 81.5 18.5
Lymph nodes enlargement	No Yes	19 10	65.5 34.5
Bone marrow biopsy	HCL infiltration Range Median	20	100 30-90 64.21
Cytopenias	Hb < 11 g/dL N < 1 × 10^9^/L PLT < 100 × 10^9^/L	6 12 18	20.6 41.4 62

**Table 2:** Clinical features of the enrolled SMZL patients.

SMZL patients (18)		n°	%
Age	Median (y) 66.8 Range (y) 56-78		
Sex	Male Female	13 5	72 28
Splenomegaly	Yes Mild Large	17 9 8	94.4 53 47
Lymph nodes enlargement	No Yes	17 1	94.5 5.5
Bone marrow biopsy	HCL infiltration Range Median	19	66.7 30-60 50
Cytopenias	Hb < 11 g/dL N < 1 × 10^9^/L PLT < 100 × 10^9^/L	8 3 10	44.4 16.6 55.5

Diagnosis of non-Hodgkin’s lymphoma was made on the basis of peripheral blood and bone marrow morphology, immunophenotype (sIg+, CD5-, CD20+, CD5/CD19-, CD25+, CD11c+, CD103+, CD10-, CD23-), clinical chemistry, imaging (chest X-ray or CT, ultrasonography of abdomen, neck, axillae, groins) or histology of suspected masses or of the spleen after surgery, according to the WHO classification 2008 (Campo et al., 2011).

On the paraffin-included samples also DBA-44 antibody has been employed in addition to the morphological analysis (Salomon-Nguyen et al., 1996).

The study was conducted according to the Helsinki declaration rules; all patients signed procedure AOUP n. 1801/T.01 February 2015 the informed consent in order to participate to the study and genomic DNA was collected and stored at the Hematology Unit of Pisa until its analysis. The only two inclusion criteria were: (1) the presence of good quality of DNA, and (2) the availability of clinical information (at diagnosis and during the follow-up).

It has to be noted that all patients included in the present study have been previously monitored by the qualitative PCR for the IgH rearrangement at diagnosis, after 3 months of treatment, and by 40 days from the end of therapy as routinary work-up.

### Treatment

Indication for treatment was represented by a neutrophil count <1 × 10^9^/L, hemoglobin <10 g/dL, platelets <100×10^9^/L, massive lymphocytosis, symptomatic splenomegaly, more than three enlarged nodes, or a history of repeated infections.

Patients affected by SMZL received oral cyclophosphamide 100 mg/day for 2 weeks every month in combination with rituximab 375 mg/m^2^ i.v. the day 8, for total six cycles, as reported by our group (Cervetti et al., 2016).

Among HCL patients, 26 received rituximab in combination with cladribine, and three alpha-interferon, at dose of three MUI three times a week.

Cladribine was infused at 0.12 mg/kg per 5 days/month for 6 months in patients aged <65 years or at 0.14 mg/Kg each week for total six consecutive weeks in older subjects.

### Clinical Response Assessment

Complete remission was defined as the absence of HCL lymphocytes in peripheral blood and in bone marrow after microscopy observation, no palpable splenomegaly or lymph nodes >2 cm, neutrophils ≥1.5 × 10^9^/L, platelets ≥100 × 10^9^/L and hemoglobin ≥11 g/dL.

Partial response required ≥50% reduction in the circulating HCL cells or spleen enlargement, and at least a ≥50% improvement of the peripheral blood values over baseline.

Progressive disease (PD) required appearance of eventual pathological lymph nodes, a ≥ 50% increase in the spleen size or a ≥50% increase in circulating HCL cells.

Stable disease was defined as lack of CR, PR, or PD.

### DNA Extraction

DNA extraction from 12 mL of bone marrow anti-coagulated with EDTA was performed using the automatic apparatus BioRobot EZ1, the EZ1 DNA Blood Card, and the EZ1 DNA Blood 350 l L Kit (Qiagen^®^, Valencia, CA, USA).

The extracted DNA was maintained at 2–8°C and quantitated using the Thermo Scientific NanoDrop 2000 spectrophotometer (Thermo Fisher Scientific^®^, Wilmington, DE, USA).

### Qualitative PCR for the IgH Rearrangement

Qualitative PCR for IgH rearrangement has been performed by fluorescent PCR, as previously described by our group (Galimberti et al., 1999; Cervetti et al., 2004).

### *B-RAF* V600E Mutation and Real-Time PCR (ARMS PCR)

Molecular analysis of the *B-RAF* V600E mutation was performed by real-time PCR (QT-PCR) using the Amplification Refractory Mutation System (ARMS) technology, which is based on the discrimination by Taq polymerase between matched and mismatched primers specifically designed for amplifying the mutated target DNA. The QT-PCR was performed on the ABI Prism 7900 HT apparatus (Applied Biosystems^®^, Foster City, CA, USA) with the *qBiomarker Somatic Mutation PCR Assay* (BRAF_476 #SMPH001828AR, Qiagen^®^, Valencia, CA, USA). All samples and controls were amplified and analyzed in duplicate, according to the manufacturer’s instructions.

The data analysis was based on the “ΔΔCt” method: the amount of mutant DNA was calculated by the formula: “ΔCt sample” = Ct of mutated allele – Ct of wild-type copy number; “ΔCt healthy donor” = Ct of mutated allele – Ct of wild-type copy number.

“ΔΔCt” = ΔCt sample – ΔCt healthy donor. A mutation call can be made when ΔΔCt > 4; when ΔΔCt is < 3 the sample was considered as wild-type; when ΔΔCt was between 3 and 4, the specimen resulted borderline.

Moreover, samples were defined as unmutated if Ct of the mutated allele was >37 (total cycles of amplification = 40).

For the relative quantitation, at each time point 2^-ΔΔCT^ in respect of sample at diagnosis was calculated.

### *B-RAF* V600E Mutation and dd-PCR

*B-RAF* V600E mutation was detected by using the specific dd-PCR BioRad assays (#10031249 assay for wild-type and #10031246 assay for mutated amplifications). dd-PCR was performed using the QX100 platform (BioRad^®^, Hercules, CA, USA), consisting of two instruments: the droplet generator and the droplet reader. The droplet generator divides the sample by creating about 20,000 partitions (droplets). The droplets are then transferred into PCR plates and, at the end of the amplification cycles, placed into the droplet reader, where each droplet is read as mutated or wild-type by issuing specific fluorescence signals (FAM for the mutation and Hex for the wild-type). These signals, after being counted, are redistributed according to the Poisson’s algorithm.

### VH Sequencing

In order to test if *B-RAF*-unmutated cases could present the VH4-34 rearrangement, three samples were sequenced on an ABI PRISM 3100 (Applied Biosystems^®^, Foster City, CA, USA) using the *Sequencing Analysis Software* v 5.1. All samples were analyzed by monoplex PCR method using six different consensus primers corresponding to the Leader VH regions (Campbell et al., 1992) in combination with a JH consensus primer. Clonal products were purified by Wizard SV^®^ Gel and PCR Clean-Up System (Promega^®^, Madison, WI, USA) and sequenced using BigDye Terminator v1.1 Cycle Sequencing Kit (Applied Biosystems^®^, Foster City, CA, USA).

The sequences were analyzed by IgBlast Network (http://www.ncbi.nlm.nih.gov/igblast/) and IMGT Information System^®^(http://www.imgt.org/IMGT_vquest/) in order to identify and quantify the IGH germline homology of clones.

### Statistical Analysis

Median and mean values of mutational burden at each time point were measured and then compared with the *t*-test. Differences were considered significant at *p* < 0.05. Analyses were performed using the SPSS 22.0 software (IBM SPSS Statistics 20; IBM Corporation, NY, USA).

## Results

### Sensitivity of the Employed Molecular Techniques

The IgH rearrangement evaluation by fluorescent PCR has been adopted in our center from many years as a simple, rapid and cheap method for the evaluation of the B-cell clonality in all B-cell lymphoproliferative diseases. In our hand, the sensitivity of this technique ranges from 1 × 10^-2^ to 1 × 10^-3^, according to the position of the clonal peak inside the Gaussian curve (Cervetti et al., 2004).

Differently from this assay, QT-PCR and dd-PCR, both able to assess the *B-RAF* V600E mutation, allow us giving both a qualitative and quantitative estimate of the mutational burden.

While in literature data concerning the sensitivity of the QT-PCR and the dd-PCR in HCL are few, analytic sensitivity tests have been performed at the start of the study, diluting a DNA from a HCL patient with 5% of *B-RAF* mutation in a pool of 5 wild-type DNAs (extracted from patients without hematological diseases during orthopedic surgery) in a half-logarithmic scale, from 1 × 10 to 1 × 10^-4^ dilution.

**FIGURE 1:**
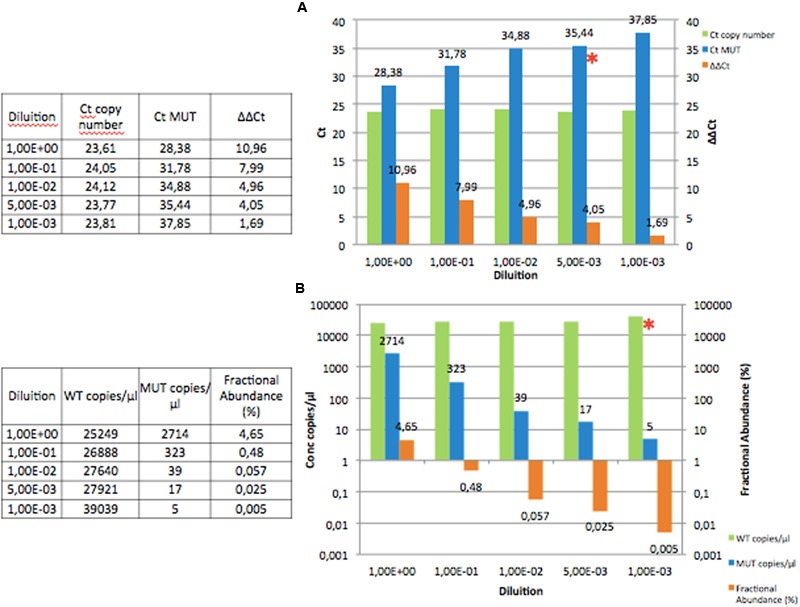
**Diluting a DNA from a HCL patient with 5% of *B-RAF* mutation in a pool of five wild-type DNAs in a half-logarithmic scale, from the 1 × 10 to the 1 × 10^-4^ dilution. (A)** In the QT-PCR the copy number Ct remained constant (green), while the mutated Ct increased (blue). In orange the trend of the ΔΔCt which is reduced up to 1.69. The 1 × 10^-3^ dilution should be considered negative, because Ct was >37 and ΔΔCt < 4.0. (See Materials and Methods) The ^∗^indicates the last dilution point that can be accepted (1 × 10^-3^). **(B)** In the dd-PCR the copy number concentration remained constant (green), while the mutated concentration reduced (blue). In orange the trend of the fractional abundance (%) which reduced up to 0.005. The ^∗^indicates the last dilution point that can be accepted (1 × 10^-3^). Ct. cycle threshold; MUT, mutated; WT, wild-type

The QT-PCR allowed detecting the *B-RAF* V600E mutation up to 5x10^-3^; considering that undiluted DNA corresponded to a mutational burden of 5%, the sensitivity of QT-PCR thus resulted of 2.5 × 10^-4^ (**Figure 1A**).

On the other hand, dd-PCR still detected mutation at 1 × 10^-3^, that corresponds to a sensitivity of 5 × 10^-5^; thus, dd-PCR showed a sensitivity of more than half a log higher than that offered by QT-PCR (**Figure 1B**).

### Assessment of the IgH Clonality on Diagnostic Samples

In order to demonstrate a clonal lymphocytic infiltration in all samples, we performed flow cytometry with assessment of κ and λ clonality and then amplified the IgH rearrangement, either in patients affected by HCL or in those with SMZL.

As expected, all the 18 cases with SMZL, all 27 patients affected by “classic” HCL and the two cases with v-HCL showed an evident IgH clonality.

### QT-PCR and dd-PCR for the *B-RAF* V600E Mutation on Diagnostic Samples

First of all, we started to test the presence of the *B-RAF* V600E mutation; when we used the QT-PCR, all 18 SMZL cases resulted unmutated, as expected. The same negative results were obtained in the two patients affected by the v-HCL.

In the remaining 27 “typical” HCL cases, the *B-RAF* mutation was found in 24 patients (88.8%).

Thus, we sequenced the VH rearrangement in the three unmutated samples; in one of them the rearrangement resulted VH4-34, thus confirming data from the literature where this kind of VH rearrangement is characterized by the absence of the *B-RAF* V600E mutation in “classic” HCL (Schnittger et al., 2012).

Thus, after discharging from the computation the two v-HCL patients and the case with the VH4-34 rearrangement, QT-PCR was able to identify the *B-RAF* V600E mutation in 24/26 patients (92.3%).

Then, the same DNA were used for the dd-PCR runs; even with this technique, the two cases with v-HCL and the VH4-34 patient resulted unmutated, so confirming the same rate of detectability already obtained by the QT-PCR (92.3%).

### Clinical Outcome and Follow-Up

After the demonstration that the *B-RAF* mutation was a specific marker of HCL also in our series, we employed either QT-PCR or dd-PCR for the assessment of MRD in the 24 cases with the molecular marker at diagnosis.

After 3 months of treatment, 9 patients achieved the CR (37.5%), 14 (58.3%) the PR, and one (4.2%) was not responding to treatment (overall response rate = 95.8%).

At the second time point (the end of treatment), all cases responded, 17 patients with CR (70.8%) and 7 with PR (29.2%).

At the 12th month, 9 patients were re-assessed: 7 were in CR (4 have previously been in PR), and 2 progressed (from PR).

### The IgH and the *B-RAF* V600E Mutation as Markers of MRD

At the first evaluation (third month of treatment), nine patients achieved the CR, 14 a PR, and one was not responsive to treatment.

A clonal IgH rearrangement was still detectable in 18 out of the 24 tested cases (75%). In particular, the B-cell clonality was still present in 13 out of the 14 cases in PR (93%), in the patient with stable disease, and in 4 of the 9 cases in CR (44.4%).

When QT-PCR was performed, the *B-RAF* V600E mutation was still detectable in 16 out of the 24 tested cases (66.6%). In particular, *B-RAF* mutation was still present in 12 out of the 14 cases in PR (85.7%), in the patient with stable disease, and in 3 of the 9 cases in CR (33.3%).

Then, dd-PCR was done: the *B-RAF* mutation was still detectable in 18 out of the 24 tested cases (75%). In particular, the mutation was still present in 11 out of the 14 cases in PR (78.6%), in the patient with stable disease, and in 6 of the 9 cases in CR (66.6%).

At the second time point (the end of treatment), 17 patients were in CR and 7 in PR.

A clonal IgH rearrangement was still detectable in 10 out of the 24 tested cases (41.7%). In particular, the B-cell clonality was still present in all cases in PR (100%), and in 3 of the 17 cases in CR (17.6%).

When QT-PCR was performed, the *B-RAF* V600E mutation was still detectable in 11 out of the 24 tested cases (45.8%). In particular, mutation was still present in 5 cases in PR (71.4%), and in 6 of the 17 cases in CR (35.3%).

Then, dd-PCR was also performed: the *B-RAF* mutation was still detectable in 13 out of the 24 tested cases (54%). In particular, mutation was still present in 5 of the 7 cases in PR (71.4%), and in 8 of the 17 cases in CR (47%).

At the month +12, 9 cases *B-RAF*-mutated at the end of treatment have been re-assessed: 7 were in CR (4 have previously been in PR), and 2 progressed from a PR.

Of the seven patients in CR, three still resulted MRD-positive by the IgH rearrangement (42.8%), and four still retained the *B-RAF* mutation (57.1%), either by QT-PCR or by dd-PCR (see **Table 3**). It is interestingly to observe that, even if still mutated, patients in CR reduced the mutational burden of more than one log in respect of the mutational burden measured at the end of therapy. In particular, the four cases coming from PR to CR significantly reduced the mutational burden, from a median value of 0.40 to 0.05%.

**Table 3:** Comparison among qualitative PCR for IgH rearrangement, QT-PCR for B-RAF V600E mutation, and dd-PCR.

	Percentage of patients with a molecular marker		
	IgH (%)	QT-PCR (%)	dd-PCR (%)
Diagnosis	100	96.4	96.4
3 months (total)	64	60	72
SD	100	100	100
PR	93	85.7	85.7
CR	30	20	50
End of treatment (total)	36	48	52
PR	100	87.5	87.5
CR	11	27.7	33.3
+ 12 months
CR	42.8	57.1	57.1

*Percentages of cases having a molecular marker are reported at diagnosis, after 3 months of treatment, and at the end of therapy.*

**FIGURE 2:**
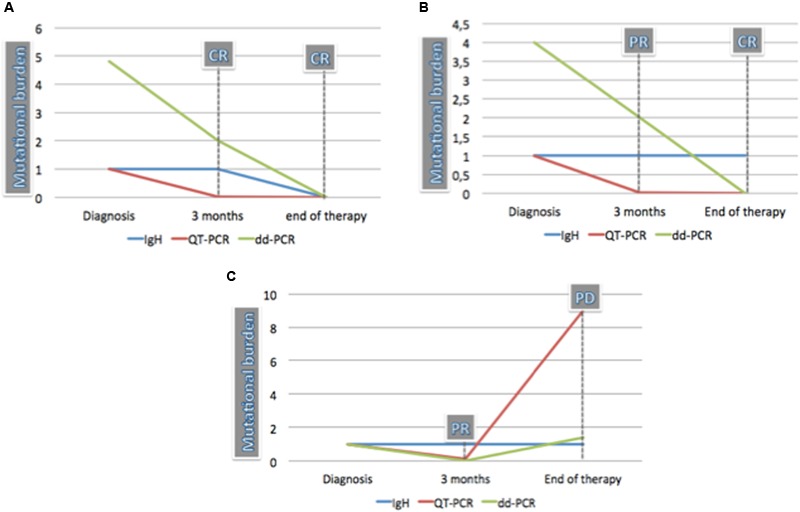
**Comparison of MRD in three HCL cases by qualitative PCR for IgH rearrangement, QT-PCR, and dd-PCR. (A)** Patient achieved the CR after 3 months and maintained it at the end of treatment. QT-PCR was negative already at the first point when dd-PCR and IgH rearrangement were positive. The mutational burden measured by dd-PCR reduced from 4.87 at diagnosis to 2 at 3 months and became negative at the end of treatment. IgH rearrangement also became negative at the end of therapy. **(B)** Patient achieved the PR after 3 months and the CR at the end of treatment. The IgH rearrangement remained clonal also when CR was achieved. The mutational burden measured by dd-PCR reduced from 4 at diagnosis to 2 at 3 months when response was partial. At this time point, QT-PCR was already negative. **(C)** Patient achieved the PR after 3 months but progressed at the end of treatment. IgH was positive both at 3 months and at the end of therapy. QT-PCR mutational burden reduced at the first time point and increased from 0.1 to 9.15 at the sixth month. The mutational burden measured by dd-PCR decreased from 1 at diagnosis to 0.01 at 3 months and increased up to 1.4 at the end of treatment.

Two patients progressed: in the first case the mutational burden increased from 2.12 to 2.45%, and in the second one from 0.01 to 1.36% (see **Figure 2**).

### dd-PCR and Quantitative Assessment

In order to verify if the quantitation of the *B-RAF* V600E mutation by dd-PCR could significantly correlate with the clinical status (thus representing a valid tool for MRD evaluation), we performed the *t*-test among the three groups: (1) cases at diagnosis; (2) cases in PR; (3) cases in CR.

At diagnosis, the mean ± standard deviation of the *B-RAF* V600E mutation was 6.82% ± 3.31%; in the group of patients in PR, it was 0.88% ± 1.18%, and in patients who achieved the CR it amounted to 0.047% ± 0.13%.

**FIGURE 3:**
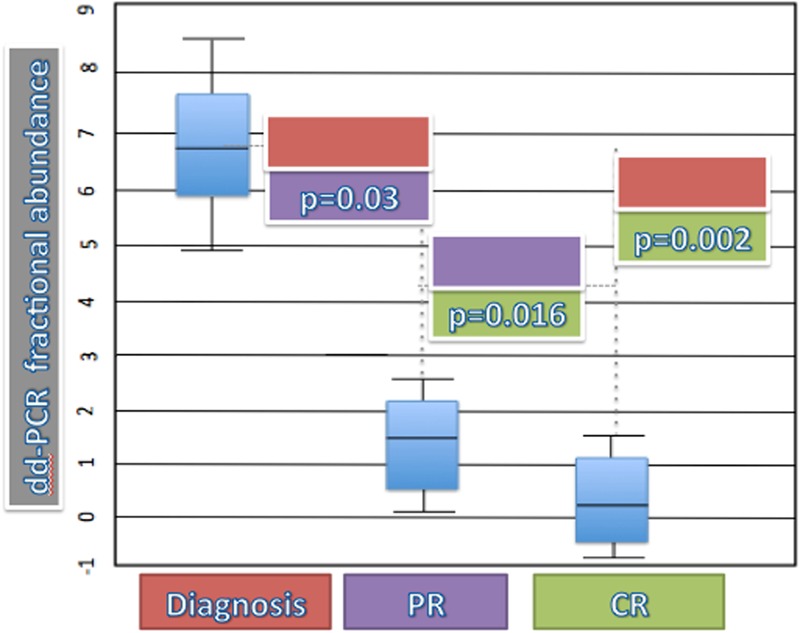
***T*-test for dd-PCR fractional abundance and status of disease.** Diagnosis (red box) vs. PR (violet box): *p* = 0.03; diagnosis (red box) vs. CR (green box): *p* = 0.002; PR (violet box) vs. CR (green box): *p* = 0.016

After statistical analysis, differences among three groups were significant: diagnosis vs PR: *p* = 0.03; diagnosis vs. CR: *p* = 0.002; PR vs. CR: *p* = 0.016 (**Figure 3**).

## Discussion

In the more recent years, the “targeted” therapy has became a reality even in hematology: in the chronic myeloid leukemia, the management of patient and the eventual switch to a second or third tyrosine-kinase inhibitor is leaded by the assessment of the *BCR-ABL1/ABL1* ratio and by the demonstration of mutations in the tyrosine domain of *ABL1*, as defined by the European Leukemia Network guidelines (Baccarani et al., 2013).

In mantle cell lymphoma, the Nordic group clearly demonstrated the predictive role of the MRD assessed by ASO-PCR, either in the setting of the conventional therapy or of the high-dose treatment (Pott et al., 2010).

In the follicular lymphoma, it has been reported that the PET-negativity in association with the molecular MRD-negativity is predictive of longer PFS (Luminari et al., 2016), with the possibility of avoiding the rituximab maintenance for patients at low risk of progression but, on the other hand, the necessity of a further consolidation in cases still PET-positive and/or MRD-positive (FIL FOLL12 trial is now being performed to confirm this hypothesis - EUDRACT NUMBER: 2012-003170-60).

In the meantime, the “pre-emptive” therapy (4 weekly doses of rituximab in patients still molecularly MRD-positive) has been shown to be efficacious to avoid relapses in follicular lymphoma (Ferrero et al., 2013), and probably this strategy could be useful in other indolent B-cell malignancies, including HCL.

With these premises, the idea of establishing already at diagnosis a patient-specific therapeutic project is now a reality; nevertheless, it is obvious that this approach needs a disease-specific molecular marker.

As occurs for *BCR-ABL1* in the chronic myeloid leukemia, the IgH clonality is a well-recognized universal marker for B-cell lymphomas, but the qualitative tests are not very sensitive and the quantitative ones need the design of patient-specific primers, with consequent lot of job and costs, and the possibility of missing an eventual emerging new clone carrying a different VH rearrangement (van der Velden et al., 2003; Della Starza et al., 2014).

In the specific setting of HCL, the discovery of the *B-RAF* V600E mutation as specific molecular marker opened the possibility of using this mutation both at diagnosis and during the follow-up for the MRD assessment.

In the present study, we applied to 18 cases affected by SMZL and 29 cases with HCL two different molecular techniques to detect the *B-RAF* V600E mutation: the QT-PCR and the dd-PCR. Both methods allowed us identifying the presence of the *B-RAF* mutation and the quantitative estimation of the mutational burden, with an obvious advantage with respect of the qualitative IgH assessment, on the basis of results coming from the follicular lymphoma (Galimberti et al., 2014), that the quantitative MRD evaluation would be more predictive than the qualitative one. Moreover, no data about dd-PCR in HCL have been previously reported in literature.

Schnittger et al. (2012) reported a valid QT-PCR method for identifying and quantitating the *B-RAF* V600E mutation. They applied this technique to 117 HCL cases and 102 control samples (patients affected by myeloid or other B and T-lymphoid malignancies), demonstrating that the QT-PCR, with sensitivity between 10^-4^ and 10^-5^, was able to detect the *B-RAF* mutation in 98.3% of tested cases. All 16 v-HCL included in the study resulted unmutated, analogously to the two cases with a “classical” HCL, but with the VH4-34 rearrangement. In the same paper, 16 patients were followed during treatment: in patients achieving a stable CR the mutational burden decreased up to 2 logs, whereas in the relapsed case it did not decrease after treatment. The authors concluded that the QT-PCR for *B-RAF* V600E mutation represented a valid approach, either at diagnosis or during the follow-up of HCL patients (Schnittger et al., 2012).

Our present study went to the same conclusion: the sensitivity of our QT-PCR was comparable to that of the German group (2.5 × 10^-4^); at diagnosis, our method detected the *B-RAF* mutation in 92.3% of cases. This percentage is 6% lower than that reported by Schnittger et al. (2012): we found in our series the same two unexplainable unmutated cases (no v-HCL, no VH4-34 rearrangement) of the German group, but in a smaller total number of cases.

Analogously to that already reported in the German paper, the specificity of our QT-PCR was optimal, because no cases with v-HCL or SMZL resulted *B-RAF* mutated.

Nevertheless, the real innovation of our study was the introduction of the innovative dd-PCR: in respect of “conventional” PCRs, dd-PCR has got the great advantage of avoiding the plasmidic reference curve, while it allows an absolute quantitation of the mutational burden, as occurs for the QT-PCR. Moreover, its sensitivity resulted optimal (5x10^-5^), as previously shown in our experience on myeloid neoplasias (Fontanelli et al., 2015).

As above reported, dd-PCR is entering now in the diagnosis and follow-up of several hematological neoplasias (Camus et al., 2016; Della Starza et al., 2016; Ommen, 2016). Thus, we considered that also the HCL could be a good field where to apply the dd-PCR.

In the present experience, dd-PCR resulted comparable to the other PCR techniques: at diagnosis, it allowed to identify the *B-RAF* mutation in 92.3% of cases with respect to the 100% of the IgH rearrangement. Nevertheless, as discussed above, IgH is not a specific marker for HCL, and so it cannot be used in the differential diagnosis from other indolent lymphomas. On the contrary, dd-PCR is a sensitive and specific technique, useful also in the diagnostic phase, where usually molecular biology is used in association with the flow cytometry.

About the possibility of using dd-PCR for MRD assessment, at the first time point, in the subgroup of CR patients (where MRD is relevant), dd-PCR was able to classify as still MRD-positive two cases already defined as MRD-negative by qualitative PCR and three of those resulted unmutated after QT-PCR. Effectively, dd-PCR missed the molecular marker in two cases showing the IgH clonality; nevertheless, these patients were in PR, condition where the MRD is less significant.

Moreover, the advantage in terms of MRD detection offered by the dd-PCR was present also at the second time points: at the end of treatment, dd-PCR identified as MRD-positive two of cases in CR resulting MRD-negative by the QT-PCR and five cases already defined as negative by the qualitative PCR for the IgH rearrangement.

Moreover, dd-PCR is interesting also from a technical point of view, because (1) it does not need a plasmidic reference curve, with the clear advantage of a minor contamination risk, and (2) it is easier to perform than the ASO-PCR, because it does not need the design of patient-specific primers; (3) the costs of dd-PCR are superimposable to those of QT-PCR, as we already showed in our previous experience in myeloproliferative neoplasias (Fontanelli et al., 2015).

Finally, as already reported by the German group for the QT-PCR, in our study also the dd-PCR well correlated with the clinical outcome: patients achieving PR or CR showed a great precocious reduction of the mutational burden, opposite to cases who progressed (where the *B-RAF* mutational burden progressively increased). The achievement of the CR was characterized by a reduction of the mutational burden of more than one log, as already reported even in the German paper (Schnittger et al., 2012).

Moreover, our statistical analysis showed that the mean of mutational burden significantly correlated with the clinical status.

## Conclusion

Our study supports the introduction of dd-PCR in the molecular assessment of HCL patients, both at diagnosis and during the follow-up. It could be clinically relevant considering that in the next future the availability of the new anti-CD20 monoclonal antibodies and of further anti-*B-RAF* compounds could be used on the basis of the “dynamic” molecular marker (alias mutational burden) monitoring.

## Author Contributions

All authors participated to the conception of the study. FG, MP, EC, SG, SS, GE, MM, MDR, IP performed PCR assays. SG, FG, LI, GC, NC, MR, GC, enrolled patients, performed flow cytometry analysis, treatment, and clinical follow-up. SG and FG performed statistical analyses. All authors approved the final version of the manuscript.

## Conflict of Interest Statement

The authors declare that the research was conducted in the absence of any commercial or financial relationships that could be construed as a potential conflict of interest.

## Abbreviations

ARMS PCR: Amplification Refractory Mutation System PCR
ASO-PCR: allele-specific-oligonucleotide PCR
CR: complete remission
dd-PCR: digital droplet PCR
HCL: hairy cell leukemia
IgH: heavy chain of immunoglobulins
miRNA: micro RNA
MRD: minimal residual disease
PR: partial response
QT-PCR: quantitative real-time PCR
SMZL: splenic marginal zone lymphoma
v-HCL: hairy cell leukemia variant
WHO: world health organization
